# Proteasome condensates repeatedly “contact and release” at the nuclear periphery during dissolution

**DOI:** 10.17912/micropub.biology.001553

**Published:** 2025-03-04

**Authors:** Conner Butcher, Kyle VanderVen, Jianhui Li

**Affiliations:** 1 Biomedical Engineering and Science, Florida Institute of Technology, Melbourne, Florida, United States

## Abstract

Stress-induced proteasome condensates have been identified in both yeast and mammalian cells. The biochemical properties and dynamics of proteasome condensates mainly depend on the specific stress conditions. In the budding yeast *Saccharomyces cerevisiae* , cytoplasmic proteasome condensates assemble from the nuclear proteasomes under glucose starvation conditions. Proteasome condensates rapidly dissipate, and proteasomes reimport to the nucleus within minutes upon glucose recovery. We characterize the kinetics and dynamics of proteasome condensates after glucose recovery. Proteasome condensates transiently associate nuclear membranes with a repetitive “contact and release” movement during dissolution. Our study provides new insight into the events leading to biomolecular condensate dissolution.

**Figure 1. Kinetics and dynamics of proteasome condensate dissolution at the indicated times in fixed and live cells:**
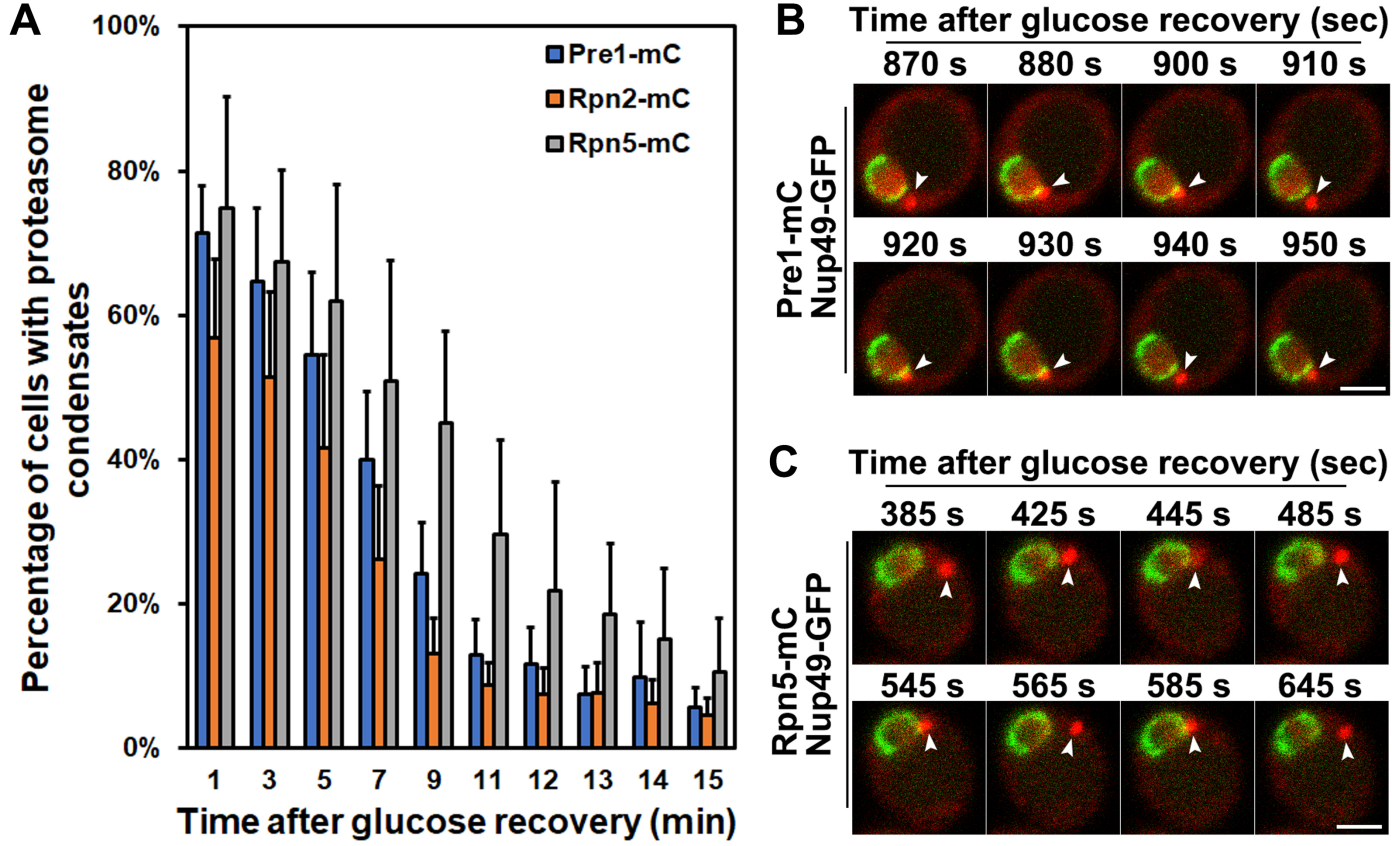
**(A)** Quantification of proteasome condensates in yeast cells at the indicated time points after glucose recovery. Yeast strains expressing a mCherry-tagged CP subunit Pre1 (Pre1-mC), lid subunit Rpn5 (Rpn5-mC), or base subunit Rpn2 (Rpn2-mC) were grown under glucose starvation conditions for four days. The glucose-starved cells were recovered and fixed at the indicated times after glucose recovery. The percentages were average numbers of five independent experiments. More than 400 cells were counted for each treatment. Results plotted as mean±sd. min: minute. **(B, C)** Confocal time-lapse live-cell images of Pre1-mC (B) and Rpn5-mC (C) in yeast strains expressing a GFP-tagged nuclear pore protein Nup49 (Nup49-GFP) after glucose recovery at the indicated time points. The figure panel (B) was selected from 27 frames of time-lapse images collected with 10-second intervals between each frame. The figure panel (C) was selected from 30 frames of time-lapse images collected with 20-second intervals between each frame. Nup49-GFP indicates the nuclear membrane. White arrowheads point to proteasome condensates. sec or s: second. Scale bars: 2 µm.

## Description

Biomolecular condensates are micro-scale membraneless compartments driven by liquid-liquid phase separation of proteins and nucleic acids, which organize spatiotemporal biomolecules and carry out efficient and dynamic cellular processes (Banani et al., 2017) . In the budding yeast *Saccharomyces cerevisiae* , hundreds of reversible condensates are assembled upon nutrient starvation at the stationary phase (Narayanaswamy et al., 2009) . Reversible cellular condensates are a hallmark feature of cellular response to increasing cell fitness under stress conditions. In contrast, dysregulation of condensates is associated with the pathogenesis of human diseases, such as neurodegenerative diseases, cancer, and viral infections (Wang et al., 2021) . Cellular condensates usually take a shorter time to dissolve than to form (Narayanaswamy et al., 2009) , and condensate dissolution is relatively less studied and understood than their formation. Previous studies have identified different pathways for disassembling condensates in the context of specific stress conditions and biomolecules. Recently, *in vitro* studies have shown that phase-separated model droplets of bovine serum albumin swim toward solvent conditions that reduce interfacial tension and favor their dissolution. This indicates that external factors, such as solvent conditions, play a role in their dissolution (Jambon-Puillet et al., 2024) . Besides external conditions, the internal biophysical and biochemical properties of biomolecular condensates also impact their dissolution. For instance, a recent theory and numerical simulation study suggests that elastic condensates are less prone to dissolution due to the bulk stress generated by elasticity (Meng and Lin, 2023) . In mammalian cells, heat-shock stress-induced specific ubiquitylation is essential to disassemble stress granules when normal growth conditions resume (Maxwell et al., 2021) . A mechanistic understanding of cellular condensate regulation will provide insight into developing novel strategies and therapeutic targets for human disease treatment.

The 26S proteasome is a large multiprotein complex comprising the core particle (CP) and the regulatory particle (RP). The RP is composed of the lid and the base subcomplexes (Bard et al., 2018) . Proteasomes are essential for selective protein degradation to maintain protein homeostasis in cell health (Rousseau and Bertolotti, 2018) . Under stress conditions, proteasomes undergo dynamic phase transitions and nucleocytoplasmic trafficking. Depending on the specific stress conditions, reversible proteasome condensates can be induced in the nucleus and the cytoplasm. Amino acid deprivation and hyperosmotic stress induce nuclear proteasome condensates in mammalian cells (Uriarte et al., 2021; Yasuda et al., 2020) . Glucose starvation conditions induce cytoplasmic proteasome condensates or proteasome storage granules (PSGs) in yeast cells, in which proteasomes export from the nucleus to the cytoplasm and assemble into reversible condensates. PSGs dissipate, and proteasomes reimport to the nucleus within minutes upon glucose recovery (Laporte et al., 2008) . Previous studies have identified multiple cellular factors regulating the dynamic proteasome phase transition and nucleocytoplasmic trafficking. For example, the AMP-activated protein kinase (AMPK) complex is necessary for proteasome condensate dissolution (Li et al., 2019) . Other cellular factors, such as cytosolic pH (Peters et al., 2013) , ubiquitin (Gu et al., 2017) , endosomal sorting complex required for transports (ESCRTs) (Li and Hochstrasser, 2022) , shuttle factors Rad23, Dsk2, Ddi1 and ubiquitin chains (Waite et al., 2024) , are involved in proteasome condensate formation under specific stress conditions. Proteasome condensates transiently associate with the insoluble protein deposit (IPOD) during formation and dissolution (Peters et al., 2016) . Although proteasome phase transitions and nucleocytoplasmic trafficking are highly regulated in cells, the dynamics and kinetics of proteasome condensate dissolution have not been well characterized.

To visualize proteasome nucleocytoplasmic trafficking and phase transitions under changing glucose conditions, we used a genetic approach to generate three yeast strains expressing fluorescent markers. These included a GFP-tagged nuclear pore protein Nup49 (Nup49-GFP) to indicate the nuclear membrane and mCherry (mC) tagged proteasome subunits. We chose to include a CP subunit Pre1 (Pre1-mC), a lid subunit Rpn5 (Rpn5-mC), and a base subunit Rpn2 (Rpn2-mC), respectively, to indicate proteasome location (Li et al., 2019) . The resulting yeast strains were glucose starved to induce proteasome condensate formation with subsequent glucose recovery to examine the kinetics of proteasome condensate dissolution in the time-course experiments. We fixed yeast cells with 2% formaldehyde, imaged the cells with a single focal plane using fluorescence microscopy at the indicated times in a 15-minute window after glucose recovery, and quantified the percentage of yeast cells with proteasome condensates containing Pre1-mC, Rpn5-mC, and Rpn2-mC, respectively. Our results showed that the percentage of cells with proteasome condensates rapidly reduced within 10 to 11 minutes after glucose recovery and then displayed a small wave pattern with a slow reduction from 11 to 15 minutes, especially in Pre1-mC and Rpn2-mC strains ( Figure 1A ). The wave reduction pattern at cell population levels indicates an active and dynamic movement of proteasome condensates after glucose recovery. Proteasome condensates may move out of and then return to the focal plane in some cells. This leads to a nonlinear trend of proteasome condensate dissolution, disappearance, and appearance pattern under a microscope with a single focal plane. Due to the 15-minute glucose recovery limit, we did not see a similar wave reduction pattern in the Rpn5-mC strain ( Figure 1A ). In the Rpn5-mC strain, 10% of cells had proteasome condensates at the 15-minute time point, whereas we observed wave patterns when cells had less than 10% proteasome condensates in the Pre1-mC and Rpn2-mC strains during the 11 to 15 minutes time window ( Figure 1A ). Given a longer glucose recovery time, we may see a similar wave pattern in the Rpn5-mC strain because proteasomes move as whole protein complexes.

Using confocal time-lapse imaging, we further checked proteasome condensate dissolution in single yeast cells after glucose recovery. We found a dynamic movement pattern of proteasome condensates that repeatedly “contact and release” at the nuclear periphery ( Figure 1B and 1C). The contact site was in a specific region at the nuclear membrane. When comparing the first selected time-lapse image with the last image, the condensate sizes became smaller after each contact or association at the nuclear periphery ( Figure 1B and 1C). This active and repetitive movement pattern of proteasome condensates indicates that proteasome condensate dissolution is highly regulated, in line with previously identified kinase signaling pathways such as AMPK (Li et al., 2019) . The transient contact sites of proteasome condensates and nuclear membranes may involve specific recognition or interaction between proteasome or proteasome-associated proteins and nuclear membrane proteins or nuclear pore complexes. We used Nup49-GFP to check if any nuclear membrane microdomains were formed after glucose recovery, but we did not observe any Nup49-GFP-associated nuclear membrane microdomains under glucose recovery conditions. We propose that proteasomes reimport to the nucleus when proteasome condensates are transiently associated with the nuclear membrane.

In this study, we have determined the kinetics of proteasome condensate dissolution in a 15-minute time window after glucose recovery and revealed a repetitive “contact and release” movement pattern of individual proteasome condensate during dissolution. Our study provides new insight into the events that lead to biomolecular condensate dissolution.

## Methods

**Yeast strains and cell growth**

Three yeast strains MHY8595 ( *MATa his3-∆200 leu2-3, 112 ura3-52 lys2-801 trp1-1 NUP49-GFP::his5+ PRE1-mC::natMX* ), MHY8599 ( *MATa his3-∆200 leu2-3, 112 ura3-52 lys2-801 trp1-1 NUP49-GFP::his5+ RPN2-mC::natMX* ), and MHY8602 ( *MATa his3-∆200 leu2-3, 112 ura3-52 lys2-801 trp1-1 NUP49-GFP::his5+ RPN5-mC::natMX* ) (Li et al., 2019) were used in this study.

Yeast cells were grown overnight at 30°C with vigorous agitation in synthetic complete (SC) medium (Li and Hochstrasser, 2022) . Cells were back diluted in fresh SC medium and grown to mid-log phase. The mid-log cells were harvested and rinsed once with sterile ultrapure water. The cells were then resuspended in SC medium containing 0.025% glucose and incubated at 30°C for four days, followed by glucose recovery at the indicated times with SC medium containing 2% glucose.

**Fluorescence microscopy**

For epifluorescence microscopy, yeast cells were chemically fixed as previously with minor modifications (Li et al., 2016) . Briefly, glucose-starved cells were washed once with sterile ultrapure water and recovered in SC medium. Glucose-recovered cells were fixed with 2% formaldehyde at the indicated times and incubated at room temperature (RT) for five minutes. The fixed cells were pelleted, washed once with 0.1M KPO _4_ pH 6.5, and resuspended in 0.1M KPO _4_ pH 7.5 for imaging. The fixed cells were visualized on an Axioskop microscope (Carl Zeiss) equipped with a Plan-Apochromat 100×/1.40 NA oil DIC objective lens, a CCD camera (AxioCam MRm; Carl Zeiss), and an HBO100W/2 light source. Images were taken using AxioVision software with an auto-exposure setup. ImageJ was used to count and quantify the percentage of cells with proteasome condensates in time-course experiments.

For confocal time-lapse video recordings, glucose-starved yeast cells were washed once with sterile ultrapure water, glucose recovered in SC medium, and viewed on an LSM 880 Airyscan NLO/FCS confocal microscope with an Alpha Plan-Apochromat 100×/1.46 NA oil objective lens. Excitation was performed with an argon laser at 488 nm and a DPSS laser at 561 nm. Emission was collected in the range of 493–556 nm or 579–624 nm for GFP and mCherry imaging, respectively. Images were acquired using ZEN software and processed using Adobe Photoshop CC software.
